# Antioxidative, Antiproliferative and Antimicrobial Activities of Phenolic Compounds from Three *Myrcia* Species

**DOI:** 10.3390/molecules23050986

**Published:** 2018-04-24

**Authors:** Catarina dos Santos, Renan S. Galaverna, Celio F. F. Angolini, Vania V. A. Nunes, Luiz F. R. de Almeida, Ana L. T. G. Ruiz, João E. de Carvalho, Regina M. T. Duarte, Marta C. T. Duarte, Marcos N. Eberlin

**Affiliations:** 1 Department of Biological Sciences, Faculty of Sciences and Letters, University of São Paulo State (UNESP), Assis 19806-900, Brazil; vaninha.nunes@gmail.com; 2 ThoMSon Mass Spectrometry Laboratory, Institute of Chemistry, University of Campinas (UNICAMP), Campinas 13083-970, Brazil; renann_galaverna@hotmail.com (R.S.G.); celio.fernando@gmail.com (C.F.F.A.); mneberlin@gmail.com (M.N.E.); 3 Department of Botany, Institute of Biosciences of Botucatu, UNESP—Univ. Estadual Paulista, Botucatu 18618-970, Brazil; luizfernando@ibb.unesp.br; 4 Faculty of Pharmaceutical Sciences, University of Campinas (UNICAMP) P.O. Box 859, Campinas 13083-859, Brazil; ana.ruiz@fcf.unicamp.br (A.L.T.G.R.); carvalho@fcf.unicamp.fcf (J.E.d.C.); 5 CPQBA, Microbiology Division, P.O. Box 6171, University of Campinas, Campinas 13083-970, Brazil; renataduarte@uol.com.br (R.M.T.D.); mduarte@cpqba.unicamp.br (M.C.T.D.)

**Keywords:** *Myrcia* spp., phenolic compounds, biological activities, ESI(−)Q-TOF-MS

## Abstract

*Myrcia bella* Cambess., *Myrcia fallax* (Rich.) DC. and *Myrcia guianensis* (Aubl.) DC. (Myrtaceae) are trees found in Brazilian Cerrado. They have been widely used in folk medicine for the treatment of gastrointestinal disorders, hemorrhagic and infectious diseases. Few reports have been found in the literature connecting their phenolic composition and biological activities. In this regard, we have profiled the main phenolic constituents of *Myrcia* spp. leaves extracts by ESI(−)Q-TOF-MS. The main constituents found were ellagic acid (*M. bella*), galloyl glucose isomers (*M. guianensis*) and hexahydroxydiphenic (HHDP) acid derivatives (*M. fallax*). In addition, quercetin and myricetin derivatives were also found in all *Myrcia* spp. extracts. The most promising antioxidant activity, measured by 2,2-diphenyl-1-picrylhydrazyl (DPPH) scavenging activity, was found for *M. fallax* extracts (EC_50_ 8.61 ± 0.22 µg·mL^−1^), being slightly less active than quercetin and gallic acid (EC_50_ 2.96 ± 0.17 and 2.03 ± 0.02 µg·mL^−1^, respectively). For in vitro antiproliferative activity, *M. guianensis* showed good activity against leukemia (K562 TGI = 7.45 µg·mL^−1^). The best antimicrobial activity was observed for *M. bella* and *M. fallax* to *Escherichia coli* (300 and 250 µg·mL^−1^, respectively). In conclusion, the activities found are closely related to the phenolic composition of these plants.

## 1. Introduction

The Myrtaceae family is one of the most important genera of the wet tropics, including South America, Australia and Tropical Asia. In Brazil, there are 23 genera and approximately 1034 species distributed throughout all regions of the country. Their leaves and berries are commonly used in traditional medicine and foods. The chemical compositions of plants belonging to the Myrtaceae family (such as *Eugenia* sp., for example) have been previously studied [1]. Myrtaceae fruits have found commercial use, for example, *Eugenia uniflora* L. (“pitangueira”), *Syzygium* sp. (“jambo”), and *Psidium guajava* L. (guava), among others. *Myrcia* is one genus of the Myrtaceae which includes shrubs and small trees, which are described an important source of essential oils, in which mono- and sesquiterpenes are predominant. The non-volatile compounds isolated from *Myrcia* spp. extracts are usually flavonoids, tannins, acetophenone derivatives and triterpenes, which have already been described to possess hypoglycemic, anti-hemorrhagic and antioxidant activities [2].

The search for phytochemicals has been increased due to their potential use in therapy as antioxidants or anticancer drugs. Among them, phenolic compounds form the major group. The importance of the search for natural antioxidants is highlighted by their action in disposing, scavenging, and suppressing the formation of reactive oxygen species (ROS) or in opposing their actions. Experimental studies have also associated oxidative cellular damage arising from an imbalance between free radical generation and scavenging systems as the primary cause of cardiovascular disease, cancer and aging. They exert different properties such as reducing agents, hydrogen donors, free radical scavengers, singlet oxygen quenchers and metal chelators [2]. 

The scavenging of DPPH radicals is widely used for rapid evaluation of the antioxidant activity of different compounds, and the inhibitory effect of tannins and flavonoids against the DPPH radical is well established [3,4]. Some of the phenolics found in Myrtaceae have already been described as scavengers of free radicals and antioxidants [3,5].

Some phenolics found in plants are also an alternative therapy against conventionally resistant infections or as new antiseptic agents. Infectious diseases are of great interest in the scientific community because some microorganisms cause severe morbidity and can be lethal. It should be noted that some Myrtaceae extracts, specifically those of *M. tomentosa*, have already been analyzed as antimicrobial agents [6].

Typically, polyphenolics and their derivatives from plant extracts are being determined, mainly *via* chromatographic protocols [7]. Direct infusion electrospray ionization high resolution mass spectrometry (ESI-HRMS) has been used for this purpose, because does not require exhaustive sampling preparation for analysis. This method allows the identification of complex organic mixtures without prior extraction or separation steps. It is fast, versatile, and sensitive, consumes a low volume of solvent and does not use more elaborate sample preparation protocols which are usually required for chromatographic separation. We have already applied ESI-HRMS with success in the phenolic fingerprinting studies of *Stryphnodrendon obovatum* extracts [1].

In this context, we report herein the rapid characterization of the main phenolic compounds from the hydroethanolic extracts of three different Myrtaceae leaves: *Myrcia bella* Cambess., *Myrcia fallax* (Rich.) DC. and *Myrcia guianensis* (Aubl.) DC., using Electrospray Ionization Quadropole Time-of-Flight Mass Spectrometry in negative ion mode (ESI(−)-Q-TOF-MS). A correlation between the compounds identified by ESI(−)-MS and the antioxidant, antiproliferative, and antimicrobial activities was also established after in vitro analysis.

## 2. Results and Discussion

**Figure 1:**
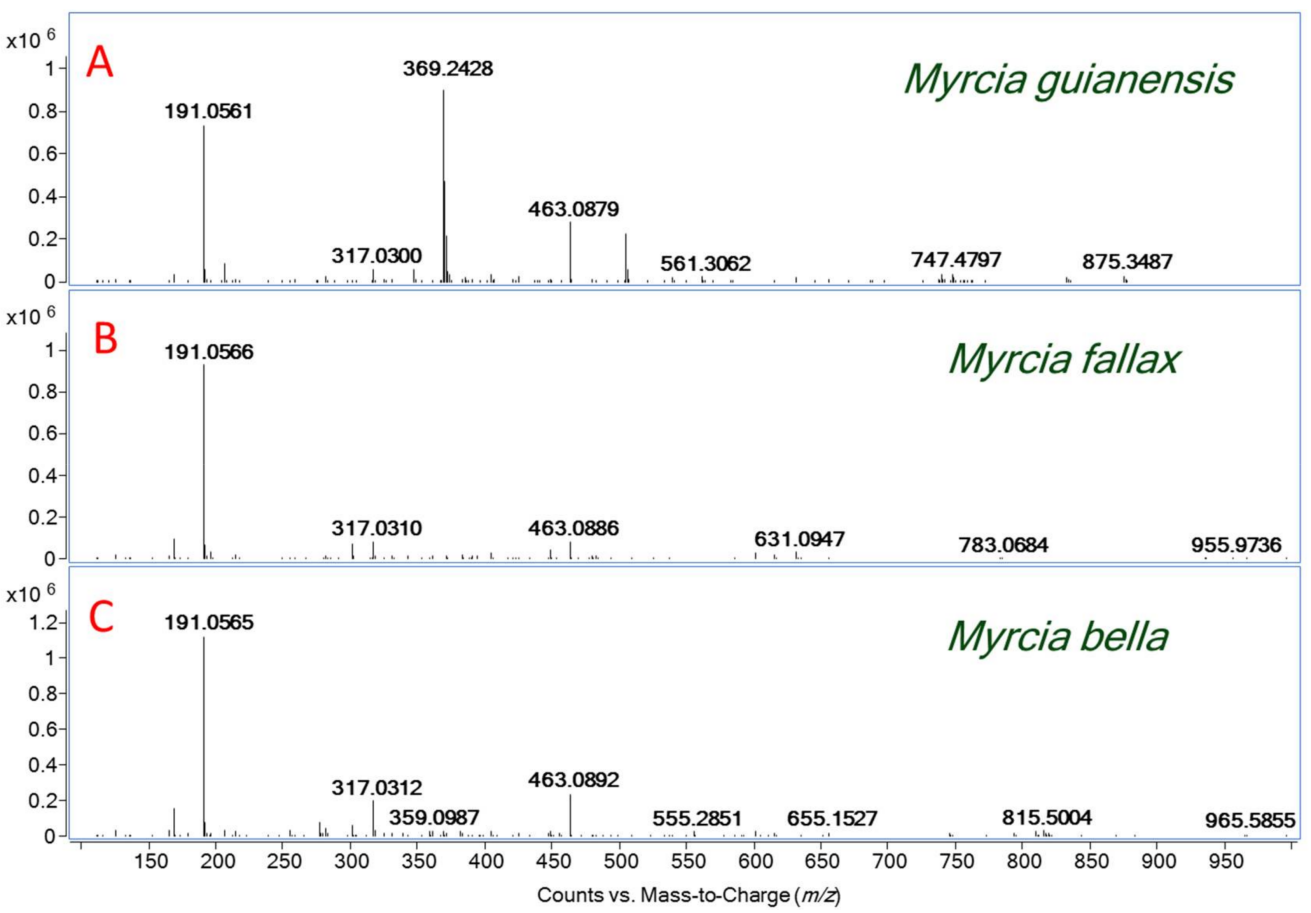
(**A**) ESI(−)-MS of the *M. guianensis* leaves extract; (**B**) ESI(−)-MS of the *M. fallax* leaves extract; and (**C**) ESI(−)-MS of the *M. bella* leaves extract.

Firstly, this research shows the main polyphenolic compounds identified from *Myrcia bella* Cambess., *Myrcia fallax* (Rich.) DC. and *Myrcia guianensis* (Aubl.) DC. Although basically the same set of ions was detected for those plants, it is clear from visual inspections that there are chemical profile differences (Figure 1). Table 1 shows the main phenolic acids and hydrolysable tannins identified via their molecular formula and dissociation patterns from all of these extracts by ESI(−)-MS/MS. Table 2 shows the main flavonols identified from these extracts. 

The compounds presented in Table 1 were identified as phenolic acids and hydrolysable tannins (gallotannins and ellagitannins), indicated by the presence of deprotonated molecular ions which correspond to gallic acid, galloyl-glucose, ellagic acid, hexahydroxydiphenic acid (HHDP) and hexahydroxydiphenoyl-glucose (HHDP-glucose). 

Phenolic acids were found in all tested *Myrcia* extracts. The first compound, gallic acid (*m/z* 169), has characteristic fragments of *m/z* 151 [M − H − H_2_O]^−^ and 125 [M − H − CO_2_] [7,8]. This fragmentation pattern is commonly found in hydroxybenzoic acid derivatives [9]. The deprotonated molecule of *m*/*z* 191 was identified as quinic acid. This compound is a primary metabolite, which can be found in great amounts in all plants and fruits [10]. The MS/MS experiment shows fragments of *m*/*z* 173 and 111, which correspond to the loss of the water molecule and a retro Diels-Alder (RDA) ring opening mechanism [10], respectively. Quinic acid has already been found in other plants of the genus Myrtaceae [11].

**Scheme 1:**
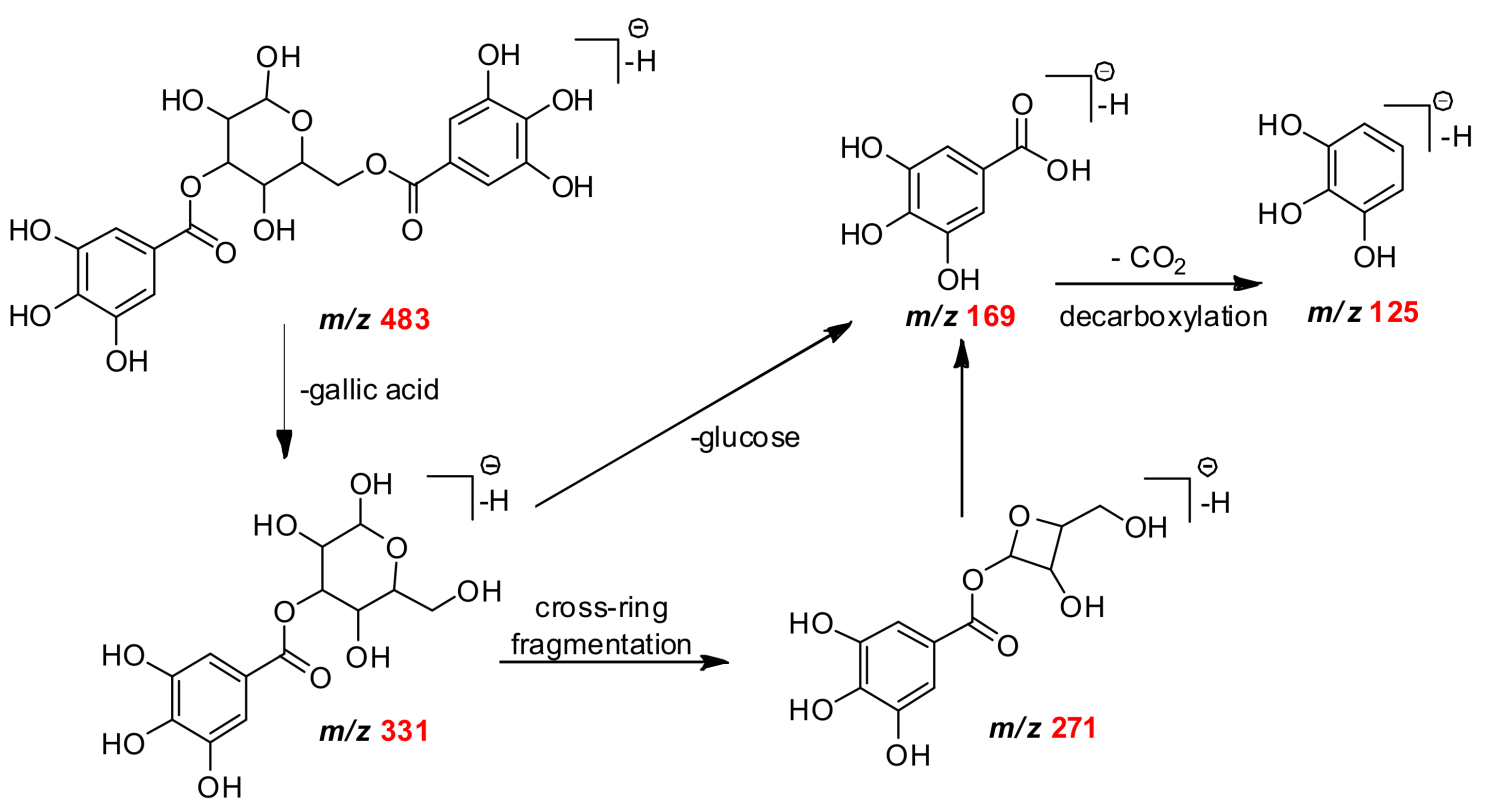
The proposed fragmentation pathways of digalloyl-glucose isomer.

Ellagic acid was characterized by the diagnostic mass ion of *m*/*z* 301 and its mass fragments of *m*/*z* 257 and 229 [12]. Monogalloyl-glucose (*m*/*z* 331) presented fragments of *m*/*z* 271, *m*/*z* 169 and *m*/*z* 125 (Scheme 1) [13,14]. The ion *m*/*z* 343 was identified as monogalloylquinic acid and MS/MS fragmentation showed loss of the galloyl moiety of *m*/*z* 191, a deprotonated gallic acid of *m*/*z* 169 and its fragment of *m*/*z* 125 [M − H − CO_2_]^−^ [14]. 

The HHDP derivatives are found mostly in *M. fallax* (Table 1). HHDP-glucose deprotonate ion molecule of *m*/*z* 481, which was followed by an ion fragment of *m*/*z* 301 (ellagic acid) after the loss of one unit of glucose. After the spontaneous lactonization of HHDP, forming ellagic acid, a subsequent decarboxylation yields an ion of *m*/*z* 275 [M − H − 44]^−^ [15,16]. The deprotonated ion molecule of *m*/*z* 483 was assigned to digalloylglucose, since it provided the typical MS/MS fragment ions of *m*/*z* 331 [M − H − 152]^−^ and *m*/*z* 169 [M − H − 162]^−^ (Scheme 1) [15]. Ellagic acid, galloyl-glucose and HHDP-glucose isomers are described in other Myrtaceae species, such as *Eugenia punicifolia* and *E. aurata* [11].

**Scheme 2:**
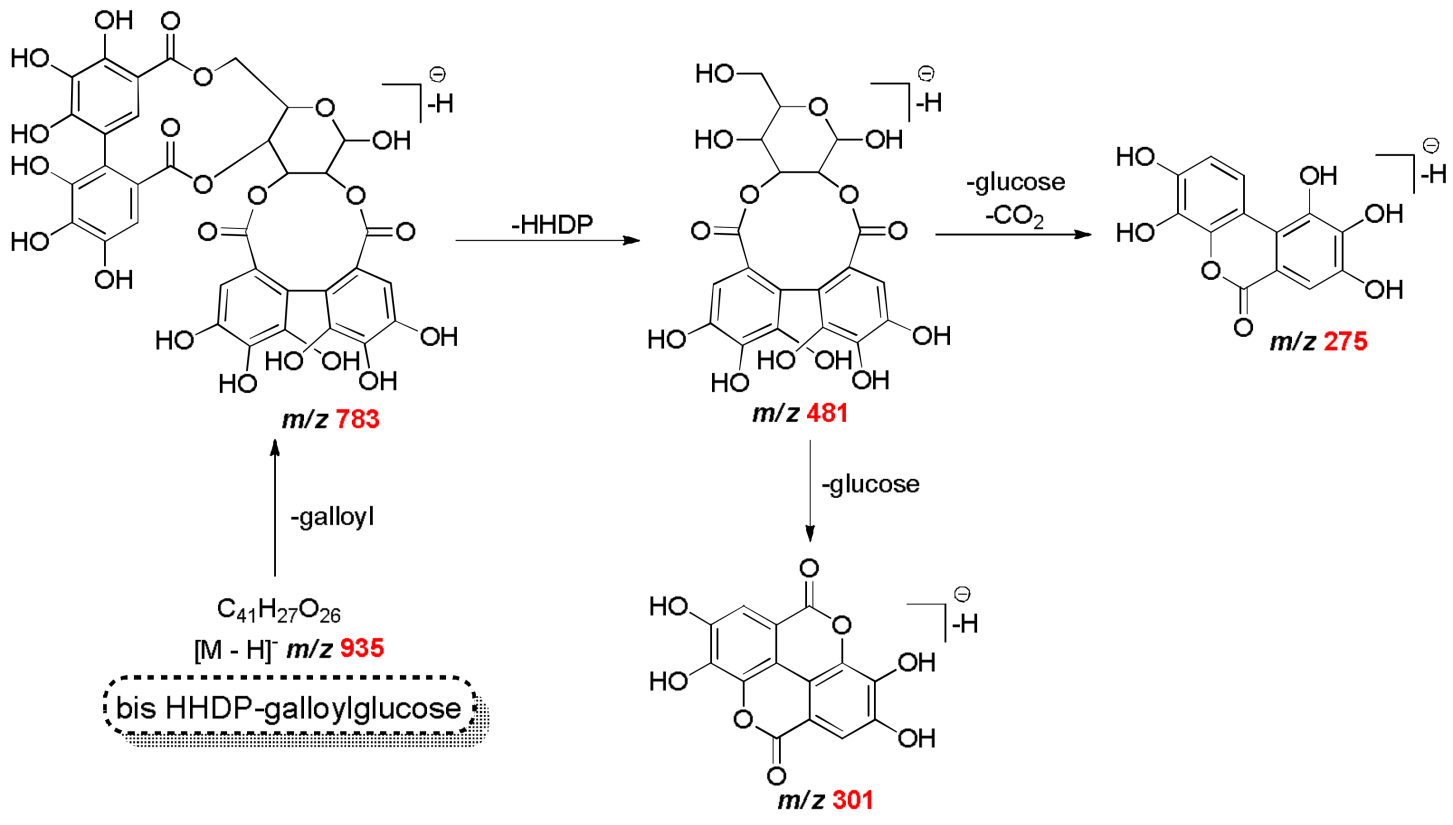
The proposed fragmentation pathways of galloyl-HHDP-glucose.

The ion of *m*/*z* 783 was assigned to bis-HHDP-glucose and the ion of *m*/*z* 935 was assigned to bis-HHDP-galloylglucose [16,17]. In the Scheme 2, the proposal for the fragmentation of these two compounds is provided. The compound detected of *m*/*z* 1085 with a fragment ion of *m*/*z* 783 corresponded to the loss of ellagic acid [M − H − 302]^−^ and *m*/*z* 301 was assigned to eucalbanin A or its isomer cornussin B (Scheme 2). Eucalbanin A and cornussisn B have been previously found in leaves and fruits of Eucalyptus species, but have never been reported in *Myrcia* spp. 

The flavonoids identified from *Myrcia bella, M. fallax* and *M. guianensis* are mostly flavonol-*O*-glycosides as quercetin, myrcetin and kaempferol. The most common sugar units linked to them are galactose, glucose, xylose and rhamnose [2]. The analyzed flavonols from in this work are presented in Table 2.

In Table 2, it can be seen that the ions of *m*/*z* 301 and *m*/*z* 317 were identified as quercetin and myricetin aglycone, respectively. Quercetin and myricetin derivatives were also described for *M. splendens, M. palustris* DC., and *M. uniflora.* Quercetin was also found in *M. myrtillifolia* [2].

Quercetin showed fragmentation ions of *m*/*z* 257 after decarboxylation [M − H − CO_2_]^−^ and of *m*/*z* 179 after cleavage of the B ring by a Retro Diels-Alder mechanism (RDA). It was also observed, after loss of a carbonyl group from fragment of *m*/*z* 179 and ion of *m*/*z* 151(^1,3^A^−^ fragment) [18,19]. Those fragmentations were also observed in myricetin, with an additional typical fragment ion of *m*/*z* 137 of the trihydroxylated B-ring [20]. 

The deprotonated molecule ion of *m*/*z* 433 was assigned to quercetin-3-*O*-D-xylopyranoside and ions products of *m*/*z* 300, 271 and 151. An ion of *m*/*z* 300 was due to the neutral loss of pentoside. From *m*/*z* 300, a CO loss was also detected to give an *m*/*z* 271 [Y_o_ − H − CO − H]^−^ and an ion of *m*/*z* 151 [19,21]. The ion of *m*/*z* 447 was assigned to deprotonated quercetin-3-*O*-β-rhamnoside and showed an aglycone fragment ion of *m*/*z* 301 [M − H − 146]^−^ due to the loss of a sugar moiety, whereas the fragment ion of *m*/*z* 271 was also typical for a flavon-3-*O*-monoglycoside [21]. 

The deprotonate molecule of *m*/*z* 449 [M − H]^−^ produced a fragment of *m*/*z* 316 [M − 132 − 2H]^−^ after loss of pentoses, as well as the fragments of the product ions *m*/*z* 179, [M − H − CO − H_2_O]^−^ and of *m*/*z* 271, confirming the presence of myricetin-3-α-*O*-arabinoside. For the ion of *m*/*z* 463, its MS/MS produced a deprotonated aglycone, that is, the ion of *m*/*z* 317 [M − H − 146]^−^, indicating that such ion is a myricetin-3-*O*-rhamnoside. Both were already described for *Myrcia* spp. [2,22]. 

The MS/MS fragmentation of *m*/*z* 479 produced the product ion of *m*/*z* 316 [M – 162 − 2H]^−^, after the loss of a glucose moiety and the product ion *m*/*z* 271 [M − H − CO − H_2_O]^−^ and *m*/*z* 179 [21]. This fragmentation pattern confirms the presence myricetin-3-*O*-β-glucoside. The deprotonate molecule of *m*/*z* 585 produced of *m*/*z* 301 [M − 132 − 152 − H]^−^, *m*/*z* 433 [M − 152 − H]^−^ and of *m*/*z* 191 from elimination of the quinic acid. Such results were in agreement with the presence of the compound quercetin-*O*-(*O*-galloyl)pentoside [22]. Myricetin-*O*-(*O*-galloyl)-arabinoside was assigned to the deprotonated molecule of *m*/*z* 601 [M − H]^−^. The MS/MS spectrum shows the ion product ion *Y*_0_^−^ of *m*/*z* 449 [M − 152 − H]^−^ due to the loss of the galloyl-glycoside moiety.

The deprotonate molecule of *m*/*z* 615 was characterized as myricetin rhamnopyranoside. Its MS/MS produced the ion of *m*/*z* 463 [M − H − 152]^−^ and the deprotonated myricetin of *m*/*z* 317 [M − H − 146]^−^ after loss of one and then two galloyl rings, respectively [22]. The fragmentation of the identified ion of *m/z* 631 [M − H]^−^ produced the ion product *m*/*z* 479 [M − 152 − H]^−^_,_ which produced the product ion product of *m*/*z* 316 [M − 152 − 16 − 2H]^−^. This compound was characterized as myricetin galloyl galactoside [23]. MS/MS fragmentation of *m*/*z* 639 [M − H]^−^ produced the ion product *Y*_1_^−^ *m*/*z* 447 [M − 192 − H]^−^ and *Y*_0_- ion of *m*/*z* 300 [M − 146 − 2H]^−^ which was characterized to quercetin-3-*O* galloyldeoxipentose. Myricetin galloyl rhamnopyranoside was assigned after fragmentation of the ion of *m*/*z* 655 which produced the fragments of *m*/*z* 463 [after loss of the galloyl moiety M − 192 − H]^−^ and of *m*/*z* 316 [M − 2H − 146]^−^.

The different extract profiles, shown in Table 1 and Table 2, affected the results for total antioxidant capacity, measured by the DPPH scavenging method (Table 3). In this work, *M. falllax* extracts showed the lowest EC_50_ value (8.61 ± 0.22 µg/mL), corresponding to the highest scavenging activity of DPPH radicals (Table 3). A possible explanation for the higher *M. falllax* activity is its own phytochemical composition, in which phenolic acids and hydrolysable tannins were found which were not present in other *Myrcia* spp. extracts. It is also remarkable that *M. guianensis* extract showed a similar EC_50_ value (16.2 ± 0.80 µg/mL) to *M. bella* (EC_50_ 13.8 ± 0.53 µg/mL), as this plant showed a much lower phenol content compared to the other extracts. A good Pearson’s correlation was found between the flavonoid content and the EC_50_ value for the DPPH antioxidant assay (r 0.774, *p* = 0.0144). The total phenolic content of Myrcia extracts did not correlate with the EC_50_ (r 0.0997, *p* = 0.799), so these results suggest that not all phenolic compounds are primarily responsible for the antioxidant activity in the DPPH. On the literature, *Myrcia bella* leave infusion showed a better EC_50_ (7.73 μg/mL), but with greather flavonoid content compared to our studies (312.99 ± 8.60 mgQ/g_extract_) [24].

One possible explanation for the EC_50_ values shown above is the synergism/antagonism of the phenolic compounds present in *Myrcia* extracts, since the antioxidant activity is usually correlated to their structures. For flavonoids, in general, structural arrangements impart the greatest antioxidant activity, since these change the ability to scavenge reactive oxygen. The activity is usually associated with the number of available hydroxyl groups in the chemical structure and delocalization of the aromatic nucleus [25]. In addition, antioxidant activity also depends on their donor-proton capacity. For example, rutin, a quercetin glycosylated molecule, decreases its own antioxidant activity [25]. Thus, quercetin and myricetin derivatives found in *Myrcia* spp. extracts satisfied all of the aforementioned determinants for antioxidant activity. The results found for flavonoids content and their correlation with EC_50_ (Table 3) were compatible with those described in the literature. From the literature, *Myrcia splendens* and *M. palustris* ethanolic extracts showed different flavonoids contents (16.43 ± 0.69 and 59.17 ± 0.53 mgQ/g_extract_) and EC_50_ (10.14 and 29.81 µg/mL), which shows that our results described above are within the range, and not that different to those of other *Myrcia* spp. [5].

As for flavonoids, the antioxidant activity of hydroxybenzoic acid and their ester derivatives also depend on the number of hydroxyl groups in the molecule. This activity is also affected by steric hindrance of the carboxylate group, since the closeness of the carboxylate group and the hydroxyl groups on the phenolic ring negatively affects their donor proton ability [2]. 

Landete described the strong correlation observed between antiradical activity measurements and the high molecular weight of hydrolysable tannins, like ellagitannins and ellagic acid. As for the other polyphenolics cited above, the major contributors to antioxidant capacity can be attributed to the presence of hydroxy functions, which exhibit a greater ability to donate a hydrogen atom and support the unpaired electron, compared to those of low molecular tannin [4]. However, as the antioxidant efficiency of these compounds is directly correlated with their degree of hydroxylation, the presence of a sugar moiety would decrease this effect. Thus, understanding the different activities of these polyphenols is critical before claiming any antioxidant-related health benefit [4]. 

The relation between cancer and oxidative stress is a “double-edged sword”. It is well-established that the over-production of reactive oxygen species (ROS) can favor chronic inflammatory processes, which are related to carcinogenesis. Thus, the three extracts of *Myrcia* spp. were also evaluated as their antiproliferative potential against a human tumor panel. We adopted the antiproliferative classification system based on Total Growth Inhibition (TGI) values described by Fouche et al. [26]. According to these criteria, a sample may be classified as inactive (TGI >50  µg·mL^−1^), weakly active (15 µg·mL^−1^ < TGI < 50 µg·mL^−1^), moderately active (6.25 µg·mL^−1^ < TGI < 15 µg·mL^−1^) and potently active (TGI < 6.25 µg·mL^−1^). Our results (Table 3) showed that the three extracts could totally inhibit the cell growth of K562 cells (leukemia) been *Myrcia guianensis* and *M. bella* extracts (TGI = 7.45 and 19.66 µg·mL^−1^, respectively), which is more active than *M. fallax* extract (TGI = 43.86 µg·mL^−1^). Moreover, *M. bella* and *M. guainensis* extracts displayed a weak selectivity activity against breast cancer (MCF-7 TGI = 30.50 and 43.32 µg·mL^−1^, respectively).

According to literature, allelopathic effects were described for *M. guainensis* leaves extracts [27]. *M. bella* leaves extract was described promoted necrosis into primary tumor gastric cell proliferation on 300 µg·mL [28]. Moreover, a US patent described a new extractive protocol to produce a *M. fallax* leaves extract with antiproliferative effect against KB cell line (human nasopharynx carcinoma) [2]. As allelopathic activity could suggest an antiproliferative effect potencial [29], our results for *M. bella* and *M. guainensis* leaves extracts are on agreement with the literature, with *M. fallax* leaves extracts with the less activity (Table 4).

Therefore, the antiproliferative effect (Table 4) observed for the three *Myrcia* species had negative correlation with the DPPH scavenger ability. These findings are on agreement with other results, showing that highly antioxidant flavonoids did not inhibit cell proliferation or polyphenolic enriched extracts [11]. Therefore, even without significant antiproliferative activity, antioxidant phytochemicals seem be useful for controlling oxidative stress in the microenvironment.

In the last decade, many Myrtaceae species have been extensively studied for their antimicrobial properties, because good activity of this genus in folk medicine has already been described. However, most of these studies, including those on *M. fallax*, have focused on essential oils from the leaves, as they are rich in sesquiterpenes and monoterpenes [2]. These terpenes are described as capable of permeating cell membranes, thanks to their hydrophobicity, which causes the death of parasites or microorganisms by affecting their metabolic pathways or organelles. For this reason, these oils could have antimicrobial activity against Gram-positive and Gram-negative human pathogens [2,30,31].

Recently, *Candida* sp. inhibition by hexane, dichloromethane, ethyl acetate and aqueous *Myrcia tomentosa* (Aubl.) DC. fractions at concentrations of 4 to 256 µg/mL was reported [6]. Those activities were previously correlated to sesquiterpenes and flavonoids.

Flavonoids are also known to be synthesized by plants in response to microbial infection, thus explaining the in vitro antimicrobial activity of those substances against a wide array of microorganisms. Their activity is probably due to their ability to complex with extracellular and soluble proteins, and lipophilic flavonoids may also disrupt the microbial membrane. From another point of view, a wide range of anti-infective actions, such as the stimulation of phagocytic cells and host-mediated tumor activity, have been assigned to tannins [31]. 

For this study, Minimum Inhibitory Concentration (MIC) values below 100 µg/mL were considered to indicate good antimicrobial activity; from 500 to 100 µg/mL to show moderate antimicrobial activity; from 1000 to 500 µg/mL to show weak antimicrobial activity; and above 1000 µg/mL to indicate inactivity [32]. Interestingly, in this study, *M. bella* and *M. fallax* have presented a moderate activity to the same Gram-negative bacteria *Escherichia coli* (300 and 250 µg/mL, respectively). *M. fallax* also showed a weak activity to Gram-positive *Enterococcus hirae* (900 µg/mL) and *M. guianensis* also presented a weak activity of 1000 µg/mL to Gram-positive bacteria *Rodococcus equi*. For all of the tests and yeast lineages there were no positive results. In the literature, *Sygyium cumini* (Myrtaceae) hydroethanolic extract has already showed a moderate activity 125 µg/mL against *Candida albicans* (ATCC 10231) yeast which was also correlated to its phytochemical content (polyphenols 100.10 mg/g extract; flavonoids 23.04 mg/g extract) [33] In this case, the antimicrobial effect of *Myrcia* spp. could be reasoned by the presence of compounds like phenolic compounds and hydrolysable tannins than galloyl glucose isomers or flavonoids.

To summarize all of the results above, the direct infusion ESI(−) QTOF-MS analysis was a helpful to provide a quite comprehensive understanding of the phenolic chemical composition of *Myrcia* spp. since they are quite different with quercetin and myricetin derivatives in common. The main constituents found which differs from one plant to another were ellagic acid (*M. bella*), galloyl glucose isomers (*M. guianensis*) and hexahydroxydiphenic (HHDP) acid derivatives (*M. fallax*). Moreover, the phenolic *Myrcia* spp. composition also showed different biological activities, which could be correlated to their chemical profiles. The most promising antioxidant activity was found for *M. fallax* Regarding in vitro antiproliferative activity, *M. guianensis* showed good activity against leukemia. The best antimicrobial activity was achieved for *M. bella* and *M. fallax* to *Escherichia coli*. In conclusion, the activities found are closely related to the phenolic composition of these plants. From these data, we could get a better insight into the unexplored pharmacological potential of those *Myrcia* spp., and further biological studies are underway in our laboratory.

## 3. Experimental Section

### 3.1. Drugs and Chemicals 

Gallic acid, quercetin, and 2,2-diphenyl-1-picrylhydrazyl (DPPH) were purchased from Sigma-Aldrich (St. Louis, MO, USA). Other reagents were purchased locally.

### 3.2. Collection and Preparation of Plant Material

Leaves of *Myrcia bella, Myrcia falax* and *Myrcia guianensis* were collected in December (2009) by Dr. Catarina dos Santos in the Instituto Florestal e Estações Experimentais—Floresta Estadual de Assis at the point 0559034/7499949 (±4 m) UTM, 0561614/7500892 (±3 m) UTM and 0561747/7501001 (±3 m) UTM Assis, State of São Paulo, Brazil (authorization process SMA: Secretaria do Meio Ambiente No. 26108-005.298/2009). *Myrcia falax* and *Myrcia guianensis* were identified by Dr. Antônio C.G. Melo, and the voucher specimen (no. 43.522 and 43.523) was deposited in the Herbarium D. Bento Pickel, Assis, SP, Brazil, for future reference. *Myrcia bella* was identified by Ms. Jorge Tamashiro and the voucher specimen (UEC 157583) was deposited in the University of Campinas (UNICAMP). For extracts, 10 g of leaves were extracted by dynamic maceration with EtOH:H_2_O 70:30 *v*/*v* (1:10 plant/solvent ratio, 3 × 2 h), at room temperature. After filtration, combined extracts were concentrated under reduced pressure until complete solvent elimination, yielding 4.40 g of *Myrcia bella* (44%), 2.38 g of *M. guianensis* (24%) and 1.9 g of *M. fallax* (19%) dry extracts.

### 3.3. Polyphenol and Flavonoid Contents

The total polyphenol content was determined in all tested extracts using Folin-Ciocalteu’s assay with some modifications [34]. Thus, to an aliquot (0.5 mL) of ethanolic solution of each extract it was added Folin-Ciocalteau reagent (2.5 mL, 10% *v*/*v*) and sodium carbonate (2.0 mL, 4% *w*/*v*) solutions, followed by thorough mixing, incubation for 120 min in the dark at room temperature and absorbance measurement at 750 nm. A calibration curve (0.5–40 µg/mL) of gallic acid was used to express the results as mg gallic acid equivalent (GAE)/g dry extract. All measurements were performed in triplicate. The total flavonoid content was determined by the aluminum chloride colorimetric assay with modifications [35]. Thus, to an aliquot (0.5 mL) of ethanolic solution of each extract, absolute ethanol (1.5 mL), aluminum chloride (0.1 mL, 10% *w*/*v*), potassium acetate (0.1 mL, 1 M) and distilled water (2.8 mL) were added, followed by thorough mixing, incubation for 30 min in the dark at room temperature and the measurement of absorbance at 425 nm. A calibration curve (5–60 µg/mL) of quercetin was used to express the results as mg quercetin equivalent (GAE)/g dry extract. All measurements were performed in triplicate.

### 3.4. ESI(−)-Q-TOF-MS Analysis

Mass spectrometry analyses were performed on an Agilent 6500 series quadrupole time-of-flight (QTOF) mass spectrometer (Agilent, Santa Clara, CA, USA), operating in the negative ion mode. The VCap voltage was maintained at 4 kV, the gas temperature was set to 290 °C and the drying gas flow rate was of 11 L·min^−1^, while the nebulizer pressure was set in 45 psi. The fragmentor was maintained at 175 V and the skimmer voltage was of 320 V. A mixture of ions with *m*/z 112.9856 and *m*/z 966.0007 was used as internal standards. Data were recorded in a mass range of *m*/z 100–1500, with a scan rate of 1 spectra s^−1^ and a MS/MS scan rate of 2 spectra s^−1^ with an isolation width of 1.3 and collision energy of 35 eV. MS/MS precursor selection was set to four precursors per cycle, where these were chosen by the abundance only mode. 

### 3.5. Determination of DPPH Radical Scavenging

The extracts for 2,2-diphenyl-1-picrylhydrazyl (DPPH) radical scavenging activity was estimated according to Enujiugha et al. with some modifications [35]. Thus, to an aliquot (2.0 mL) of methanolic solution of each extract (1.56–200 µg/mL), DPPH solution (0.5 mL, 0.03% in methanol) was added, followed by vigorous mixing, incubation for 30 min in the dark at room temperature and the measurement of absorbance at 517 nm. Absolute methanol (2 mL) was used as the blank (negative control) and quercetin and gallic acid solutions were used as positive controls. All tests were carried out in triplicate. The DPPH radical scavenging activity of each extract was calculated according to Equation (1):

(1) $$\%\;\mathrm{inhibition}\;=\;\left(1-\frac{{\mathrm{Abs}}_{\;\mathrm{sample}}}{{\mathrm{Abs}}_{\;\mathrm{control}}}\right)\times 100$$

where Abs _sample_ is absorbance of each extract and Abs _control_ is absorbance of methanol in DPPH. 

### 3.6. In Vitro Antiproliferative Activity Assay

Human tumor cell lines U251 (glioma), UACC-62 (melanoma), MCF-7 (breast), NCI-ADR/RES (ovarian expressing phenotype with multiple drugs resistance), 786-0 (renal), NCI-H460 (lung, non-small cells), PC-3 (prostate), OVCAR-03 (ovarian), K562 (leukemia) and a non-tumor cell line VERO (epithelial cells of monkey kidney) were obtained from the National Cancer Institute (Frederick, MD, USA). Stock cultures were grown in medium containing 5 mL of RPMI 1640 (GIBCO BRL, Gaithers- burg, MD, USA) supplemented with 5% fetal bovine serum (FBS, GIBCO) at 37 °C with 5% CO_2_. Penicillin:streptomycin (1000 µg·L^−1^:1000 U·L^−1^, 1 mL·L^−1^) was added to the experimental cultures. Cells in 96-well plates (100 µL cells well^−1^) were exposed to the extracts in DMSO (Sigma-Aldrich)/RPMI (0.25, 2.5, 25, and 250 µg·mL^−1^) at 37 °C, and 5% CO_2_ in air for 48 h. The final DMSO concentration (0.2% in higher concentration) did not affect cell viability. Before (T0) and after (T1) sample application, cells were fixed with 50% trichloroacetic acid (Merck) and cell proliferation was determined by the spectrophotometric quantification (540 nm) of cellular protein content using sulforhodamine B assay. Using the concentration–response curve for each cell line, the values of the sample concentration required to produce total growth inhibition or cytostatic effect (TGI) were determined through non-linear regression analysis using software ORIGIN 8.6^®^ (OriginLab Corporation, Northamptom, MA, USA) [1,11]. 

### 3.7. Minimum Inhibitory Concentration

The antimicrobial activity of the hydroethanolic extract and fractionated extracts of *Myrcia* spp. was identified by microdilution in broth, determining the minimum inhibitory concentration (MIC) and minimum fungicidal concentration (MFC) following the norms established by the Clinical and Laboratory Standards Institute (CLSI) [32]. The following microorganisms were tested: *Bacillus subtilis* (gram positive, ATCC 6051), *Enterococcus faecium* (gram positive, ATCC 5079), *Staphylococcus aureus* (gram positive, ATCC 6538) *Salmonella choleraessuis* (gram negative, ATCC 10708), *Enterococcus hirae* (gram positive, ATCC 10541), *Micrococcus luteus* (gram positive, ATCC 4698), *Rodococcus equi* (gram positive, ATCC 25729), *Streptococcus epidermidis* (gram negative, ATCC 12228), *Escherichia coli* (gram negative, ATCC 11775), *Pseudomonas aeruginosa* (gram negative, ATCC 13388) and *Candida albicans* (ATCC10231). The test was carried out in 96-well microplates containing 100 μL/well of the specific culture medium (brain heart infusion (BHI; Difco, Franklin Lakes, NJ, USA) for bacteria and RPMI 1640 (Angus Buffers & Biochemicals, Niagara Falls, NY, USA) for yeast). The substances were diluted in 40% alcohol (8 mg/mL) and transferred to the first well, before serial dilutions were performed to obtain concentrations between 15.62 and 2000 μg/mL. Chlorhexidine 0.12% (Sigma-Aldrich) and nystatin (7–8 mg/mL, Sigma-Aldrich) were used as the positive controls and 40% alcohol was used as the negative control. In addition, 40% Alcohol was tested and showed no antimicrobial activity against the microorganism studied. The bacterial inocula (colony-forming units (CFU)/mL) and fungal inocula (CFU/mL) were added to the wells and the plates were incubated at 37 °C for 24 h. The MIC was defined as the lowest concentration of the extract or fraction that inhibited visible microbial growth, which was confirmed with 0.01% 3-(4,5-dimethylthiazol-2-yl)-2,5-diphenyl-tetrazolium bromide (MTT, Sigma-Aldrich) for bacteria and by the change in color of the RPMI 1640 for yeast. All assays were performed in triplicate in three independent experiments. 

### 3.8. Statistical Analysis

The results were expressed as mean values ± standard deviation from each *Myrcia* spp. extract parameter in triplicate. Experimental data were evaluated by variance analysis (one-way ANOVA, Bioestat v 5.3, Marinauá Institute, Tefé, AM, Brazil) followed by Tukey test. A significance level of 5% was adopted. Statistical significance was set at a level of *p* < 0.05. Correlation and regression analyses were performed using ORIGIN 8.6^®^ software (OriginLab Corporation).

## Figures and Tables

**Table 1:** Phenolic acids and hydrolysable tannins identified by ESI(−)-MS from *Myrcia* spp. leaves extracts.

Formula [M − H]^−^	Theoretical Mass [M − H] *m*/*z*	Experimental Mass [M − H]^−^ *m*/*z*	∆m (ppm)	MS/MS Fragments *m*/*z*	Compound Identification	Myrcia Species
C_7_H_5_O_5_	169.0158	169.0162	2.36	151, 125	gallic acid	Mb
C_7_H_11_O_6_	191.0561	191.0562	−0.52	173, 111	quinic acid	Mb Mf Mg
C_14_H_5_O_8_	300.9987	300.9979	0.84	257, 229	ellagic acid	Mf Mb
C_13_H_15_O_10_	331.0671	331.0668	2.65	271, 211, 169, 125	monogalloylglucose	Mb Mf Mg
C_14_H_15_O_10_	343.0666	343.0660	1,75	191, 169, 125	monogalloylquinic acid	Mf
C_20_H_17_O_14_	481.0624	481.0623	0.16	301, 275	HHDP-glucose isomer	Mf
C_20_H_19_O_14_	483.0770	483.0775	−1.03	331, 169, 125	digalloyl-glucose isomer	Mf Mg
C_34_H_24_O_22_	783.0657	783.0659	−0.26	481, 301, 191	bis HHDP-glucose	Mf
C_41_H_27_O_26_	935.0708	935.0760	−5.56	783, 481, 451, 301	bis HHDP-galloylglucose (casuarinin)	Mf
C_48_H_29_O_30_	1085.0720	1085.0738	−1.66	935, 783, 301	eucalbanin A/cornusin B isomer	Mf

**Table 2:** Flavonols identified from *Myrcia* sp. leaves extracts.

Formula [M − H]^−^	Theoretical Mass [M − H] *m*/*z*	Experimental Mass [M − H]^−^ *m*/*z*	∆m (ppm)	MS/MS Fragments *m*/*z*	Compound Identification	Myrcia Species
C_15_H_9_O_7_	301.0348	301.0348	0.01	257, 179, 151	quercetin	Mf Mb
C_15_H_9_O_8_	317.0303	317.0304	−0.32	299, 179, 151, 137, 107	myricetin	Mb Mg Mf
C_20_H_17_O_11_	433.0768	433.0765	0.69	300, 271, 151,	quercetin–3-*O*-β-d-xylopyranoside	Mf Mb
C_21_H_19_O_11_	447.0927	447.0932	−0.28	316, 287, 271, 179	quercetin-3-*O*-β-rhamnoside	Mf Mg Mb
C_20_H_17_O_12_	449.0718	449.0720	−1.12	316, 271,179	myricetin-3-*O*-arabinoside	Mf Mg
C_21_H_19_O_12_	463.0877	463.0880	−0.65	317, 271, 179	myricetin-3-*O*-rhamnoside	Mf Mb Mg
C_21_H_19_O_13_	479.0821	479.0821	0.01	316, 271, 179, 151	myricetin-3-*O*-β-glucoside	Mf Mg
C_27_H_21_O_15_	585.0886	585.0877	1.54	433, 301, 191	Quercetin-*O*-(*O*-galloyl)pentoside	Mf Mb
C_27_H_21_O_16_	601.0835	601.0823	2.00	449, 316, 271, 179	Myricetin-*O*-(*O*-galloyl)arabinoside	Mf Mb
C_28_H_23_O_16_	615.0947	615.0981	0.98	463, 317, 301, 179, 151	myricetin rhamnopyranoside	Mf Mb Mg
C_28_H_23_O_17_	631.0930	631.0928	0.32	479, 316, 191, 169, 151	Myricetin galloyl galactoside	Mf Mg
C_28_H_31_O_17_	639.1567	639.1553	2.19	447, 300, 191	quercetin-3-*O*-galloyldeoxipentose	Mb
C_28_H_31_O_18_	655.1516	655.1510	0.92	463, 316, 191	myricetin galloyl rhamnopyranoside	Mb Mf Mg

**Table 3:** Antioxidant results of *Myrcia* spp. extracts measured by DPPH. Phenol and flavonoid content assay.

Reagent/Extract	Phenol Content ^a^	Flavonoid Content ^a^	DPPH (µg/mL)
	mgGAc/g_extract_	mgQ/g_extract_	EC_50_
Gallic Acid			2.03 ± 0.02 ^a^
Quercetin			2.96 ± 0.17 ^a^
*M. bella*	215.39 ± 0.01 ^a^	20.24 ± 0.48 ^a^	13.8 ± 0.53 ^b^
*M. fallax*	218.19 ± 0.81 ^a^	25.92 ± 0.50 ^b^	8.61 ± 0.22 ^c^
*M. guianensis*	71.54 ± 0.01 ^b^	19.17 ± 0.30 ^a^	16.2 ± 0.80 ^d^

^a^ Results expressed as mean ± S.E.M.; EC_50_: amount of antioxidant necessary to decrease the initial DPPH concentration by 50%; GAc: Gallic acid; Q: Quercetin.

**Table 4:** Total Growth Inhibition (TGI) values, given in µg·mL^−1^, for *Myrcia* spp. extracts and doxorubicin (DOX) necessary for total inhibition of tumor cell proliferation.

	MCF-7	NCI-ADR/RES	786-0	NCI-H460	PC-3	HT-29	K562
DOX	3.30	6.60	2.67	0.90	5.85	3.90	20.48
*M. bella*	30.50	132.48	128.69	139.82	94.23	63.58	19.66
*M. fallax*	>250	>250	245	110.95	190.78	151.11	43.86
*M. guianensis*	43.32	>250	209.83	193.29	94.73	170.34	7.45

DOX: Doxorubicin (positive control); *M. bella*, *M. fallax*, *M. guaianensis*: *Myrcia* spp. leaves extracts. Human tumor cell line: MCF-7 (breast), (ovarian expressing multiple drug resistance phenotype), 786-0 (renal), NCI-H460 (lung, non-small cells), PC-3 (prostate), HT-29 (colon), K562 (leukemia). TGI > 50 μg·mL^−1^ = inactive extract.
